# RETRACTION: Notch1 Protects Against Myocardial Ischaemia‐Reperfusion Injury via Regulating Mitochondrial Fusion and Function

**DOI:** 10.1111/jcmm.70999

**Published:** 2025-12-31

## Abstract

**RETRACTION**: DaiS.‐H. , WuQ.‐C. , ZhuR.‐R. , WanX.‐M. and ZhouX.‐L. , “Notch1 Protects Against Myocardial Ischaemia‐Reperfusion Injury via Regulating Mitochondrial Fusion and Function,” Journal of Cellular and Molecular Medicine 24, no. 5 (2020): 3183–3191, 10.1111/jcmm.14992.31975567 PMC7077547

The above article, published online on 23 January 2020 in Wiley Online Library (wileyonlinelibrary.com), has been retracted by agreement between the authors; the journal Editor‐in‐Chief, Stefan N. Constantinescu; the Foundation for Cellular and Molecular Medicine; and John Wiley & Sons Ltd. The retraction has been agreed upon following concerns raised by a third party. An investigation determined that Figures 4C, 4D, and elements of Figure 6B, duplicate figures previously published in other articles by a different author group. Due to errors in image management leading to unreliable data, the authors have voluntarily requested retraction. The editors consider the results and conclusions compromised.

**RETRACTION**: S.‐H. Dai , Q.‐C. Wu , R.‐R. Zhu , X.‐M. Wan and X.‐L. Zhou , “Notch1 Protects Against Myocardial Ischaemia‐Reperfusion Injury via Regulating Mitochondrial Fusion and Function,” Journal of Cellular and Molecular Medicine 24, no. 5 (2020): 3183–3191, 10.1111/jcmm.14992.31975567 PMC7077547

The above article, published online on 23 January 2020 in Wiley Online Library (wileyonlinelibrary.com), has been retracted by agreement between the authors; the journal Editor‐in‐Chief, Stefan N. Constantinescu; the Foundation for Cellular and Molecular Medicine; and John Wiley & Sons Ltd. The retraction has been agreed upon following concerns raised by a third party. An investigation determined that Figures 4C, 4D, and elements of Figure 6B, duplicate figures previously published in other articles by a different author group. Due to errors in image management leading to unreliable data, the authors have voluntarily requested retraction. The editors consider the results and conclusions compromised.

